# Extracting Effective Image Attributes with Refined Universal Detection

**DOI:** 10.3390/s21010095

**Published:** 2020-12-25

**Authors:** Qiang Yu, Xinyu Xiao, Chunxia Zhang, Lifei Song, Chunhong Pan

**Affiliations:** 1 National Laboratory of Pattern Recognition, Institute of Automation, Chinese Academy of Sciences, Beijing 100190, China; qiang.yu@nlpr.ia.ac.cn (Q.Y.); songlifei2018@ia.ac.cn (L.S.); chpan@nlpr.ia.ac.cn (C.P.); 2 School of Artificial Intelligence, University of Chinese Academy of Sciences, Beijing 100049, China; 3 School of Computer Science and Technology, Beijing Institute of Technology, Beijing 100081, China; cxzhang@bit.edu.cn

**Keywords:** attribute extraction, Refined Universal Detection, word tree, image captioning

## Abstract

Recently, image attributes containing high-level semantic information have been widely used in computer vision tasks, including visual recognition and image captioning. Existing attribute extraction methods map visual concepts to the probabilities of frequently-used words by directly using Convolutional Neural Networks (CNNs). Typically, two main problems exist in those methods. First, words of different parts of speech (POSs) are handled in the same way, but non-nominal words can hardly be mapped to visual regions through CNNs only. Second, synonymous nominal words are treated as independent and different words, in which similarities are ignored. In this paper, a novel Refined Universal Detection (RUDet) method is proposed to solve these two problems. Specifically, a Refinement (RF) module is designed to extract refined attributes of non-nominal words based on the attributes of nominal words and visual features. In addition, a Word Tree (WT) module is constructed to integrate synonymous nouns, which ensures that similar words hold similar and more accurate probabilities. Moreover, a Feature Enhancement (FE) module is adopted to enhance the ability to mine different visual concepts in different scales. Experiments conducted on the large-scale Microsoft (MS) COCO dataset illustrate the effectiveness of our proposed method.

## 1. Introduction

Attribute extraction is an important process in various computer vision tasks. Commonly, the attributes are defined as the probabilities of frequently-used words belonging to any part of speech (POS) corresponding to images. Recently, researchers have used attributes to strengthen the applications in image classification, facial verification, image captioning [1,2], etc. For example, attributes with high probabilities of words “man”, “food”, “eating”, and “delicious” indicate that there is probably a man who is eating delicious food in that image. It shows that the attributes containing high-level semantic information can help in understanding the content of images.

The application of attributes is of paramount importance in image captioning, which is a process of generating natural sentence descriptions for a given image based on the objects, together with their actions and relationships in the image. Recent work shows that attributes containing high-level semantic information can significantly improve the performance of caption generation [3,4]. As a computer vision task, the attribute extraction process is commonly implemented using Convolutional Neural Networks (CNNs). Since each image corresponds to multiple words, the attribute extraction can be treated as a multi-label object classification or detection task. Researchers usually attempt to use weakly supervised methods, for example, Multiple Instance Learning (MIL) [5,6], to train the CNN-based attribute detection networks [3,7].

Although recent methods attach great importance to the attributes that guide computer vision processes, the attributes they used are not effective enough. The reasons are explained as follows. First, as shown in Reference [3], taking only CNN and MIL as the structure of attribute detection, the performances on the attributes of non-nominal words, including verbs, adjectives, and others, except for nouns, are significantly worse than those of nouns. The reason lies in that CNNs behave worse in extracting features for abstract concepts than for concrete objects. Second, those methods treat synonymous nominal words as independent and different words, thus ignoring the similarities among them.

To solve the above two problems, a Refined Universal Detection (RUDet) method is proposed in this paper to improve the attribute extraction process. It mainly contains the following three modules. First, to refine the performance on non-nominal words, a well-designed Refinement (RF) module is developed. It generates refined attributes of non-nominal words based on the integrated knowledge of both the original detected attributes and visual features. In other words, the attributes of non-nominal words depend on not only the visual features but also the attributes of nominal words. Second, a Word Tree (WT) module is constructed to detect more reasonable attributes for synonymous nominal words. In the WT module, all nominal words are assigned to different levels of parent nodes according to their relevance. Using this module, the probability of one leaf word is calculated as the product of itself and its all ancestors. This mechanism ensures that similar nominal words, or called synonyms, hold similar probabilities, which are reasonable according to natural knowledge. Third, a Feature Enhancement (FE) module is employed to detect attributes of different visual concepts in different scales. The proposed method has the ability to detect various kinds of concepts, thus rendering an approach of “universal” detection. The comprehensive experiments are conducted to illustrate the effectiveness of the proposed method on extracting image attributes. By replacing the attributes used in state-of-the-art caption generation methods, the experiments also indicate the validity of our proposed method in caption generation.

Overall, the main contributions proposed in this paper are:

- An RF module refines the detected attributes for non-nominal words. It can generate refined attributes of non-nominal words with the help of the integrated knowledge of both the original detected attributes and visual features.
- A WT module improves the attributes for synonymous nouns. It can generate reasonable attributes for similar nominal words based on the prior knowledge in the word tree structure.
- An FE module is employed to extract multi-scale features of input images, which is responsible for mining different visual concepts in different scales. Furthermore, it helps to generate more accurate attributes for all words.
- Comprehensive experiments indicate that our method outperforms the state-of-the-art attribute detection methods and can improve the performance of many image captioning methods.

## 2. Related Work

Existing methods employ only CNNs as the attribute detection networks. Most image captioning methods split the task into two separate processes: attribute detection and caption generation. Those methods detect attributes using attribute detection models and map the attributes to caption sentences by language models. As a weakly supervised method, MIL learns to predict the instance probabilities, while only bag probabilities are in prior knowledge [5,6]. The structure based on CNN and MIL is commonly used in generating attributes. Fang et al. [3] implemented the attribute detection following the structure based on CNN and MIL. They picked the feature map from the last convolutional layer of backbone AlexNet [8] or VggNet [9] and calculated the instance probabilities through two convolutional layers followed by a sigmoid function. Then, the attributes are calculated as the bag probabilities of corresponding words using MIL. Yao et al. [4] adopted a similar structure using GoogLeNet [10]. The GoogLeNet goes deeper, thus getting more representative features, which improves the performance of attribute detection. Wu et al. [11] designed a region-based framework that takes a series of region proposals as input, and then detects attributes on those regions. Then, attributes from all regions are integrated using the max pooling operation.

Given the detected attributes, researchers attempted to enhance their image captioning methods. Yao et al. [4] attempted to feed the attributes into their caption generation model in different ways to prove the helpfulness of the attributes. Wu et al. [11] directly learned a mapping from the attributes to sequences of words as the caption descriptions. Yang et al. [12] designed a content module for visual features and a linguistic module for textual information. The content module takes the images as input and output the visual attributes, while the textual module takes the visual attributes as input and maps them to a frequency vector. Finally, the outputs of the two modules are integrated and then fed into the following Long Short-Term Memory (LSTM) [13] to generate captions. Rennie et al. [14] adopted the reinforcement learning method, called self-critical sequence training, to directly optimize the test metric on the image captioning dataset. They utilized the spatially pooled attribute features or the spatial attention features generated by CNN models, and fed the features into the LSTM to generate caption sentences. Anderson et al. [15] proposed a combined bottom-up and top-down attention mechanism, using the complex and powerful object detection network Faster Region-based CNN (Faster R-CNN) [16] to generate effective image features. These kinds of features are widely used in many image captioning methods later. Huang et al. [7] constructed an end-to-end model, connecting the attribute detection with the following caption generation. They designed a multimodal attribute detector, which learned a mapping from the bottom-up and top-down features to attributes corresponding to frequently-used words, and improved the performance by mutual promotion between image features and word embedding of attributes. Xiao et al. [17] fed the attributes into all steps of the following caption generation model, which helps to modulate the feature distributions of the semantic representation.

However, all the previous methods suffer from the two problems mentioned above: they cannot effectively deal with non-nominal words, and they ignore the similarities between synonymous nominal words. To solve these problems, the RUDet method is proposed in this paper.

## 3. Method

As mentioned above, the proposed RUDet method was developed to detect effective and refined image attributes. It takes the image as input, extracts the features from the visual concepts, and then outputs the attributes indicating the probabilities of the presences of *N* frequently-used words in the input image. Note that the proposed RUDet acts like a weakly supervised object detection method and has the following two properties: (1) It is responsible for detecting various kinds of “entities”, including physical objects and abstract concepts. (2) Although it can predict the coarse position of entities, the probabilities of the entities’ presences are enough.

**Figure 1:**
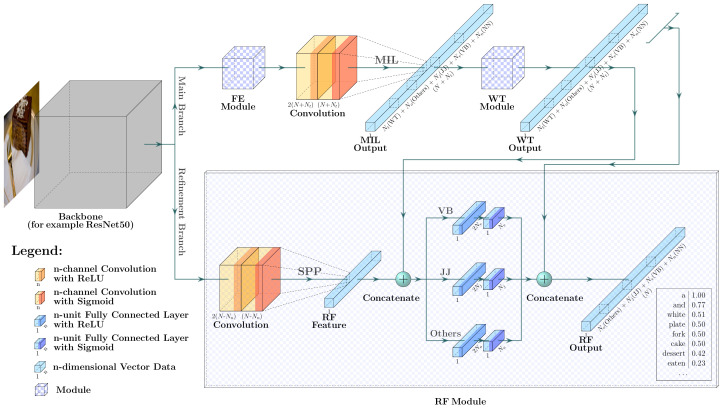
The network structure of our proposed Refined Universal Detection (RUDet). The main branch detects attributes using the Feature Enhancement (FE) module, the convolutional layers, the Multiple Instance Learning (MIL) method, and the Word Tree (WT) module. The Refinement (RF) module in the refinement branch integrates the visual features extracted by the Spatial Pyramid Pooling (SPP) layer and the attributes from the main branch, and then it learns a non-linear mapping to refined attributes for words of each non-nominal part of speech (POS), including verbs (VB), adjectives (JJ), and others.

The base model of our proposed RUDet follows the structure of “CNN + MIL”. The CNN is adopted to extract spatial visual features of images. Meanwhile, the MIL is employed as a weakly supervised training method to learn the probabilities of words’ presence in the images and to learn which regions are most likely associated with these words. The more accurate probabilities mean more effective attributes, which can better represent the semantic information of the images. In order to boost the accuracy of the detected attributes, three additional modules are proposed in our RUDet, including the RF module, the WT module, and the FE module. These modules are described in the following subsections. Figure 1 demonstrates the overall structure of our method.

### 3.1. Base Model

The base model consists of a CNN feature extraction backbone and an MIL header module. The powerful and widely used Residual Networks (ResNets) [18] are employed as the backbone network. On the output feature map of the backbone, the MIL header module is expected to learn the attributes, that is, the probabilities of *N* frequently-used words. In the MIL header module, a series of convolutional layers are employed to detect the probability of each word corresponding to each grid area. For example, given an image of size 512×512, the backbone ResNet50 and the convolutional layers output a feature map of size N×16×16, where *N* is the number of frequently-used words. In this feature map, it can be supposed that each value represents the probability of the word’s presence in the corresponding 32×32 area in the input image. Then, the MIL method is used to calculate the probabilities of words’ presences in the whole image.

MIL is a weakly supervised method which learns predicting the instance probabilities while only bag probabilities are in prior knowledge [5,6]. The Noisy-OR version of MIL [5,6] is used to calculate the probabilities of words in image sub-regions while knowing only the probabilities in the whole image. Following the implementation in Reference [3], instead of using fully connected layers, a series of convolutional layers and a sigmoid activation function are used to get the probabilities of words corresponding to regions, which are represented by the values in the convolutional feature map. For the word $w_{i}$ in the whole set of words $W=\{w_{i}|1⩽i⩽N\}$, a sample image I is labeled positive if $w_{i}$ exists in I; otherwise, it is negative. In the proposed RUDet, the backbone network and the following convolutional layers together extract the features of the input image I, which is denoted as feature map C. The shape of C is (N,H,W), where *H* denotes height, *W* denotes width, and *N* is the number of words. Each value $C_{i}^{x,y}$ in the feature map C can be mapped to a rectangle region in the input image I, where x,y is the spatial coordinate in the feature map C. Thus, $C_{i}^{x,y}$ is considered as the probability of $w_{i}$ in the corresponding region. Then, the Noisy-OR version of MIL is defined as:

(1) $$P_{i}=\mathrm{MIL}\left(C_{i}\right)=1-\prod_{x\in [1,W],y\in [1,H]}\left(1-C_{i}^{x,y}\right),\;i\in \left\{1,2,\cdots ,N\right\},$$

where $P_{i}$ stands for the probability of the word $w_{i}$ in the image I. After training from a dataset containing labels of whole images, our proposed RUDet can identify the sub-regions where the visual concepts lie in the images.

To sum up, the base model takes the image as input, extract visual features by a CNN backbone and additional convolutional layers, and then it outputs the probabilities of words as the attributes of that image. Thus, the process of the base model is formulated as:

(2) $$B=B\left(I\right),$$

(3) $$C=\mathrm{sigmoid}\left(\mathrm{CONV}\left(B\right)\right),$$

(4) $$P^{base}=\mathrm{MIL}\left(C\right),$$

where $B\left(\cdot \right)$ stands for the backbone network, $\mathrm{CONV}\left(\cdot \right)$ denotes the additional convolutional layers, sigmoid is the sigmoid activation function, and $P^{base}$ represents the attributes detected by this base model.

### 3.2. Refinement Module

Although the base model of the proposed RUDet has the ability to detect words of all POSs, it lacks accuracy in detecting non-nominal words because visual features are not enough to effectively detect abstract or invisible concepts. For example, it is easy to detect the noun “person” because it shows in an image as an exact region of pixels. However, it is hard to find a region related to non-nominal words, like “beautiful”, in an image; thus, they cannot be easily detected. The refinement branch, also named the Refinement (RF) module, was developed to improve the detection performance for non-nominal words.

We designed the RF module as a non-linear mapping function from a set of known knowledge to refined attributes of non-nominal words, where the knowledge consists of not only the image visual features but also the initial attributes from the base model. The initial attributes from the base model, or more precisely the initial attributes of nominal words, provide the most important information that guides in learning more accurate attributes of non-nominal words. Technically, a series of convolutional layers and the following Spatial Pyramid Pooling (SPP) layer [19] are employed to extract a fixed-length visual feature vector of the image. Next, it integrates the visual feature and the original attributes from the main branch, and then it learns a non-linear mapping by a series of fully connected layers to generate refined attributes for words of each non-nominal POS. The following formulas show the process of the RF module:

(5) $$C^{rf}=\mathrm{sigmoid}\left(\mathrm{CONV}\left(B\right)\right),$$

(6) $$S^{rf}=\mathrm{SPP}\left(C^{rf}\right),$$

(7) $$P_{pos}^{rf}={\mathrm{RF}}_{pos}\left(P\right)=\mathrm{sigmoid}\left({\mathrm{FC}}_{pos}\left(S^{rf}⊕P\right)\right),\;pos\in \left\{\mathrm{VB},\mathrm{JJ},\mathrm{Others}\right\},$$

where B denotes the output of the backbone network in the base model, $S^{rf}$ is the fixed-length visual feature vector from the SPP layer, P stands for the original attributes of all words from the main branch, $\mathrm{RF}\left(\cdot \right)$ represents the RF module, and $P_{pos}^{rf}$ stands for the refined attributes of words $W_{pos}$ which belong to non-nominal POS pos. Meanwhile, $\mathrm{SPP}\left(\cdot \right)$, $\mathrm{FC}\left(\cdot \right)$, and ⊕ represent the SPP layer, the fully connected layers, and the concatenation operation, respectively.

The main difference, compared with the CNN-based base model, is that the RF module contains a non-linear mapping for each non-nominal POS implemented by fully connected layers. The non-linear mapping is fed with not only the visual features but also the helpful knowledge of attributes of nominal words from the main branch. This helps the RF module to generate more accurate attributes for non-nominal words. The improvement of this module is shown in the results of the ablation study experiments in this paper.

### 3.3. Word Tree Module

Besides the issue of non-nominal words which has been addressed by the RF module, there is also a problem with the attributes for nominal words. Commonly, more than half of the *N* frequently-used words are nouns. However, there are many synonyms in these nouns. In general, synonymous words should be related to same visual concepts; thus, their probabilities should be similar in each image. In order to ensure similar probabilities for synonyms, a hierarchical Word Tree (WT) module is constructed using the $N_{n}$ nouns in all *N* words. For the remaining non-nominal words, including verbs, adjectives, and others, there are no obvious hierarchical relationships among them. Thus, there is no need for non-nominal words to be considered in the WT module.

**Figure 2:**
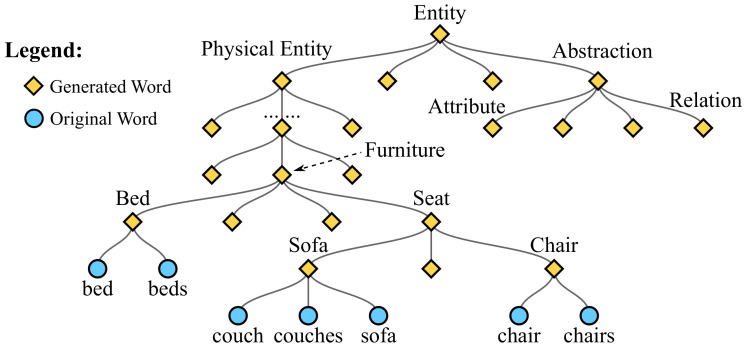
A simplified demonstration of the word tree.

In our implementation, the WT module is built based on the prior knowledge from the well-known WordNet [20], which is a lexical database of English. The WordNet helps to find conceptual relationships between words, such as hypernyms, hyponyms, and synonyms, antonyms, etc. These relationships guide the construction of the WT module to integrate similar words. Figure 2 demonstrates a simplified WT. The process of constructing the WT module is explained below. First, the root node of the WT is “entity”, which means the collect of all noun words, and the original $N_{n}$ noun words are mapped to $N_{n}$ leaf nodes in the WT. Then, the WordNet is used to find the parent path from leaf nodes to the root node. For example, “chair” and “couch” are two leaf nodes which exist in the original $N_{n}$ noun words. According to the knowledge of WordNet, both “chair” and “couch” belong to “seat”, so a node “seat” is created as their parent node. Then, “seat” and “bed” belong to “furniture”, so “furniture” is also added as their parent node in the WT. The similar processes are carried out until the root node is reached.

**Figure 3:**
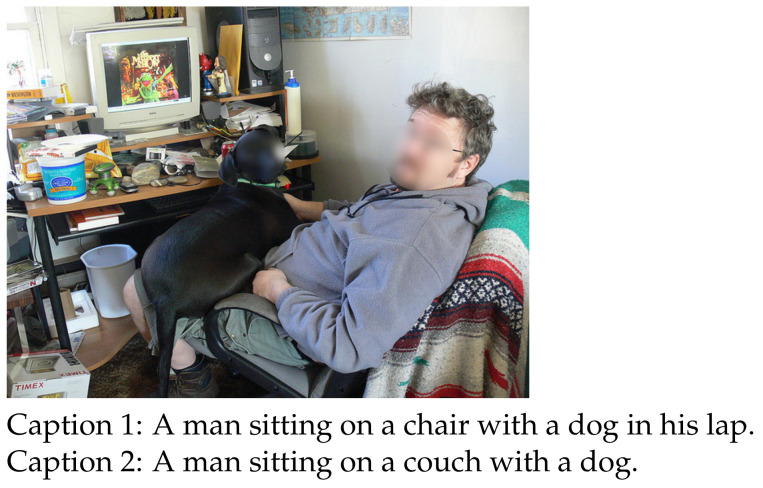
The words “couch” and “chair” are synonyms in the captions of an image.

After building the WT, other $N_{t}$ parent words are generated. These $N_{t}$ words are appended to the previous *N* words to form the complete word set of size $N+N_{t}$. In the implementation of the WT, both the input and the output are $N+N_{t}$ dimensional vectors. In detail, for each word, the input value is considered as its conditional probability on the basis of its direct parent word, and the output value is the absolute probability, which is computed as:

(8) $$P_{i}^{wt}=\mathrm{WT}\left(P_{i}\right)=P_{i}\cdot \prod_{i^{\prime }\in A_{i}}P_{i^{\prime }},\;i\in \left\{1,2,\cdots ,N+N_{t}\right\},$$

where $P_{i}$ stands for the conditional probability of the word $w_{i}$ before the WT, $P_{i}^{wt}$ represents the absolute probability after the WT, and $A_{i}$ denotes the indices set of all ancestor words of $w_{i}$. For example,

(9) $$\begin{array}{cc} P_{chair}^{wt} & =P_{chair}\cdot P_{Chair}\cdot P_{Seat}\cdot \;\ldots \;\cdot P_{Entity}, \end{array}$$

(10) $$\begin{array}{cc} P_{couch}^{wt} & =P_{couch}\cdot P_{Sofa}\cdot P_{Seat}\cdot \;\ldots \;\cdot P_{Entity}, \end{array}$$

which shows that the probabilities of similar words “chair” and “couch” are more likely to be similar, owing to the contributions from their same ancestors. Therefore, synonymous nominal words hold similar probabilities, which makes our RUDet robust to words with similar semantics. For example, the synonyms “chair” and “couch” in Figure 3 hold similar probabilities after computed through WT module.

### 3.4. Feature Enhancement Module

To make the proposed RUDet robust to visual concepts of different scales, the Feature Enhancement (FE) module is adopted to extract multi-scale features of input images. The FE module is designed based on a simplified version of the Feature Pyramid Network (FPN) [21]. The modification is that only the levels of P4, P5, and P6 are used as multi-scale feature maps to reduce the memory usage. This module generates multi-scale feature maps based on the backbone network. Then, the MIL header module described in the base model is used on these three multi-scale feature maps to simultaneously compute attributes for multi-scale visual concepts. Then, these three attributes are integrated using the weighted sum operation.

Formally, the FE module is expressed as:

(11) $$E_{4},E_{5},E_{6}=\mathrm{FP}N^{\prime }\left(B_{4},B_{5}\right),$$

(12) $$C_{k}^{fe}=\mathrm{sigmoid}\left({\mathrm{CONV}}_{k}\left(E_{k}\right)\right),\;k\in \left\{4,5,6\right\},$$

(13) $$P_{k}^{fe}=\mathrm{MIL}\left(C_{k}^{fe}\right),\;k\in \left\{4,5,6\right\},$$

(14) $$P^{fe}=\sum_{k\in \left\{4,5,6\right\}}\lambda_{k}\cdot P_{k}^{fe},$$

where $B_{4}$ and $B_{5}$ are two feature maps from the last two blocks of the backbone network [21], ${\mathrm{FPN}}^{\prime }\left(\cdot \right)$ is the simplified version of the FPN, $E_{4}$ to $E_{6}$ are three multi-scale feature maps, $P_{k}^{fe}$ stands for the attributes detected from the feature map $E_{k}$, $\lambda_{k}$ is a learnable weight, and $P^{fe}$ represents the integrated attributes which are the final output of the FE module.

### 3.5. Loss Function

The frequencies of the words are seriously unbalanced, which makes it difficult to perform well on the detection task of all words. For this reason, the class-balanced loss proposed in Reference [22] is adopted to train our model. The loss function is formulated as:

(15) $$L\left(P,Y\right)=-\sum_{i=1}^{N}\frac{1-\beta }{1-\beta^{n_{i}}}\left[P_{i}\log Y_{i}+\left(1-P_{i}\right)\log\left(1-Y_{i}\right)\right],$$

where $P_{i}$ and $Y_{i}$ represent the predicted probability and the ground truth label for the *i*-th word $w_{i}$, respectively, *N* denotes the total number of words, $n_{i}$ represents the frequency of $w_{i}$ in the training dataset, and β is a hyper parameter. It attempts to find the best balance point by assigning an effective number to each class. By assigning different factors to the loss items of words with different frequencies, this loss function can help to relieve the problem of unbalanced data.

In the proposed RUDet model with all modules, the final loss function consists of three different items, that is:

(16) $$L_{final}=L_{base}+L_{wt}+L_{rf},$$

where $L_{base}$ denotes the loss value calculated by the ground truth and the output attributes from the base model and the optional FE module, and $L_{wt}$ and $L_{rf}$ represent the loss values of the attributes from the WT module and the RF module, respectively. Because the WT module improves the attributes of nominal words only, and the RF module improves the attributes of non-nominal words only, thus, $L_{wt}$ is calculated using the nouns (NN) part of the attributes, while $L_{rf}$ is calculated using the other part, excluding NN, that is, the non-nominal part. Note that the ground truth labels of the generated parent words in the WT module can be calculated using the labels of leaf words, which can be used as an extra supervision of the WT module. In detail, the loss functions of the WT module and the RF module are defined as:

(17) $$\begin{array}{cc} L_{wt} & =L\left(P^{n},Y^{n}\right)+L\left(P^{t},Y^{t}\right), \end{array}$$

(18) $$\begin{array}{cc} L_{rf} & =L\left(P^{o},Y^{o}\right), \end{array}$$

where $P^{n}$, $P^{t}$, and $P^{o}$ stands for the NN part, the generated parent part, and the non-nominal part of the attributes, and the same as the ground truth labels $Y^{n}$, $Y^{t}$, and $Y^{o}$.

## 4. Experiments

### 4.1. Dataset

**Figure 4:**
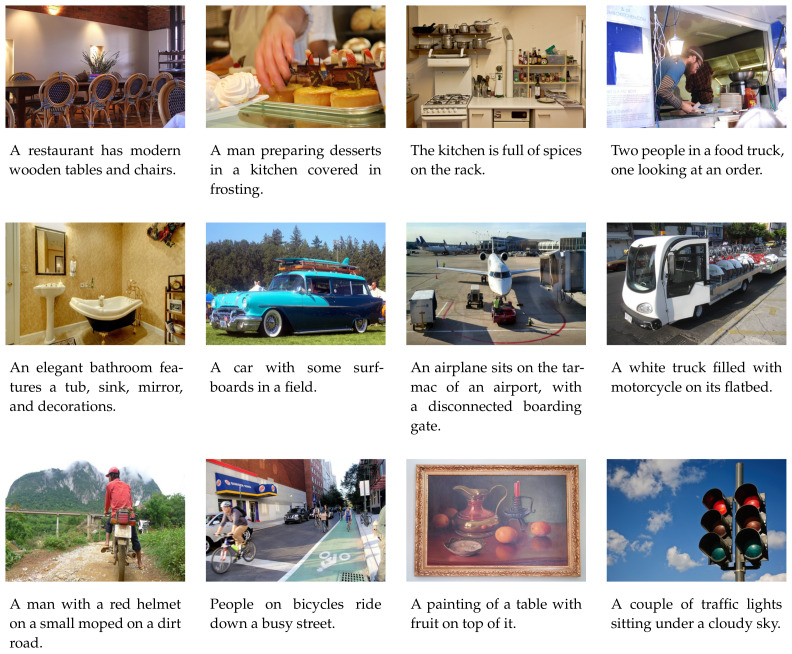
The images which are randomly chosen from the Microsoft (MS) COCO dataset. The text under each image shows the first sentence from the five ground truth caption sentences associated with the image.

The public available Microsoft (MS) COCO dataset [23] is used to conduct the experiments of training and evaluation. MS COCO is a large-scale and widely used image dataset for use of object detection, segmentation, caption generation, and so on [3,4,7,11,15,16,17,24,25,26]. It consists of 123,287 images in total. Each image is associated with at least five caption sentences annotated by humans. Sampled images and the corresponding captions are shown in Figure 4. There are various of complex visual concepts, including scenes, objects, colors, actions, and relationships, in the huge number of images. In addition, different scales, different lighting environments, and different camera poses make it a quite difficult task to detect accurate attributes from these images. Thus, the MS COCO dataset is enough for training and evaluating our method. The Karpathy’s splits (https://cs.stanford.edu/people/karpathy/deepimagesent/) are used to divide the dataset into training set, validation set, and testing set. Unless otherwise mentioned, the following experimental results are reported on the testing set.

### 4.2. Attribute Detection

#### 4.2.1. Experimental Settings

**Figure 5:**
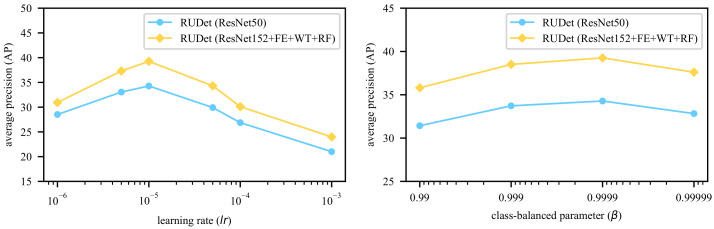
Different Average Precision (AP) values (the higher the better) with different hyperparameters, including the learning rate lr and the class-balanced parameter β.

Following the settings in References [3,7], N=1000 frequently-used words are chosen from the training caption sentences in the proposed models. The words in the ground truth sentences are used to form the labels for attributes. In the WT module, $N_{n}=616$ and $N_{t}=313$. The numbers of words belonging to other POSs are shown in Table 1. All images are resized so that the longer side contains 512 pixels. In the training phase, the Adam optimizer [27] is adopted with default parameters $\beta_{1}=0.9$ and $\beta_{2}=0.999$, which are widely used by researchers in various tasks [7,14,28]. Different values of learning rate lr are tested, and, finally, the learning rate is set to $10^{-5}$, which generates the best result. The parameter β in the class-balanced loss function is set to 0.9999, which is tested to best fit the unbalanced dataset. The experimental results for different hyperparameters are shown in Figure 5. The models are implemented based on the deep learning framework PyTorch (https://pytorch.org/). All backbones have been pre-trained on the ImageNet dataset [29].

#### 4.2.2. Experimental Results

Quantitative Results: Referring to Reference [3], the metric Average Precision (AP) for multi-label classification problems is used in the evaluation. Given a probability threshold $t\in \left[0,1\right]$, for a word *w* and an image I, if the model’s output probability is $P_{w}⩾t$, the image is considered a true positive instance if the word exists in the annotated caption sentences; otherwise, it is a false positive instance. Then, the precision and recall values are calculated by the number of true positive instances and false positive instances corresponding to different probability thresholds. Finally, the evaluation metric AP is computed as the area under the precision-recall curve by numerical integration. Four other methods are employed here to be compared to our proposed RUDet method. The “MIL (AlexNet)” and “MIL (VGG)” [3] are attribute detection methods which are implemented by directly using CNN and MIL. The “MAD” is an attribute detector which is separated from the image captioning method Multimodal Attribute Detector plus Subsequent Attribute Predictor (MAD+SAP) [7]. The AP values of “MAD” are calculated using the pretrained image captioning model from the authors (https://github.com/RubickH/Image-Captioning-with-MAD-and-SAP/). For comparison, we trained the “MAD” model separately on the attribute detection task only, which is named as “MAD-retrained” in the results. The results are reported in Table 2. It shows that “MAD-retrained” can perform better than “MAD”, which was joint trained on the image captioning task but not the attribute detection task. Compared to all these methods, the proposed method performs better in AP for all words. This experiment indicates the effectiveness of our method, showing the performance improvement on the attribute detection.

**Figure 6:**
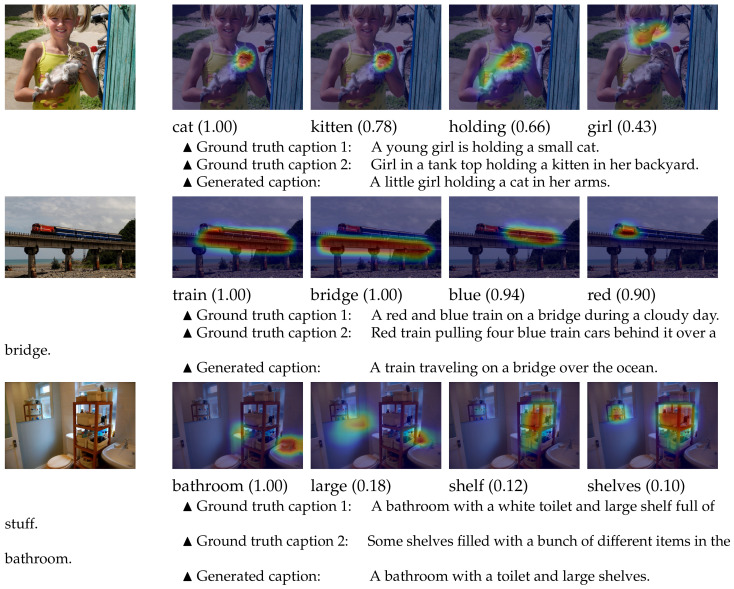
The visualization of the localization heat maps from the proposed RUDet, and the generated captions from the RUDet plus Multimodal Attribute Detector plus Subsequent Attribute Predictor (RUDet+MAD+SAP). The first column shows the original images. The following columns are the heat maps indicating the probabilities of major words using class activation mapping (CAM) [34], where red regions denote higher probabilities, and blue regions denote lower probabilities. The sentences below show the ground truth captions and the generated sentences.

Qualitative Analysis: Qualitative results are shown in Figure 6. It demonstrates that our proposed model can effectively detect words corresponding to visual concepts in images. More importantly, with the help of the RF module, non-nominal words for abstract concepts can also be correctly detected, such as the words “holding”, “blue”, and “large”. In addition, synonymous nominal words are reasonably detected with similar probabilities, for instance the words “shelf” and “shelves”, which owes to the WT module. These results indicate that our proposed method is powerful to extract attributes for words of all POSs.

#### 4.2.3. Ablation Study

In order to show the effectiveness of the modules in our method, we design five different versions of the proposed RUDet model.

- The first version, or named the base model, contains only a backbone network and a series of convolutional layers for calculating the probabilities of words corresponding to sub-regions in the image. This base model generates the initial attributes of input images. The MIL, as a weakly supervised method, is applied to calculate the probabilities of words corresponding to the whole image and to train this base model.
- To verify the effectiveness of the FE module, the second version is constructed with a backbone network and the FE module. The FE module is adopted to extract multi-scale features of input images, which is expected to generate more accurate attributes.
- To show the improvement of the WT module, the third version is built using the base model and the WT module, which helps to improve the performance on synonymous nominal words.
- To indicate the refinement effect of the RF module, the fourth version consists of the base model and the RF module, which refines the attributes of non-nominal words.
- The fifth version integrates the base model and all above modules, which is the most powerful model.

The results of ablation experiments are reported in Table 1. First, it shows that higher AP appears along with deeper backbone. This may owe to the higher-level semantic information extracted by the deeper CNNs. Second, fixing the backbone, the FE module brings the increase of the AP for all words by about 1.1. This is a considerable improvement for this issue, which benefits from the multi-scale features by the FE module. Third, the WT module and the RF module result in the AP increment of about 1.4 and about 0.9, respectively. Note that, although the RF module aims at improving the performance on non-nominal words, the APs of nominal words also get increased. These increments benefit from the joint training of the base model and the RF module, i.e., better attributes of non-nominal words have a positive impact on the training process of nominal words. This is the same reason why the WT module generate better attributes of non-nominal words, in spite of the fact that it aims at improving the performance on synonymous nominal words.

To further demonstrate the effect of the WT module on synonymous nominal words, the AP values of two examples of synonyms are reported in Table 3. The results show that the WT module reduces the differences between synonymous nominal words and, furthermore, increases the mean AP of them all. These results indicate that the WT module can ensure similar and more accurate probabilities for synonyms.

When combining all these modules, the strongest version of the proposed RUDet comes out. The integration of the deeper backbone network ResNet152, the FE module, the WT module, and the RF module achieves the increment of the AP of all words at about 5.0. This is a significant improvement, which shows that our proposed RUDet method can detect effective attributes of images.

### 4.3. Caption Generation

Additional experiments on the image captioning task were conducted to verify the advantage of the attributes detected by our method. Six state-of-the-art image captioning methods are employed for comparison, including Long Short-Term Memory with Attributes (LSTM-A) [4], Attribute Region CNN plus LSTM (Att-R+LSTM) [11], Multimodal Attribute Detector plus Subsequent Attribute Predictor (MAD+SAP) [7], Self-critical Sequence Training (SCST) [14], Up-Down [15], and Scene Graph Auto-Encoder (SGAE) [28]. In addition, we report the evaluation results of three methods, including “RUDet+LSTM-A”, “RUDet+Att-R+LSTM”, and “RUDet+MAD+SAP”, where the “RUDet+” means replacing the attributes used in the original method with the attributes detected by the proposed RUDet. The results are shown in Table 4. The values of standard metrics are reported, including BiLingual Evaluation Understudy (BLEU) [30], Meteor [31], Recall-Oriented Understudy for Gisting Evaluation - Longest Common Subsequence (ROUGE-L) [32], and Consensus-based Image Description Evaluation - Defended (CIDEr-D) [33]. Compared with their original results, the image captioning models using the detected attributes by our method achieve much higher performance. This demonstrates that the attributes detected by our proposed RUDet can represent more abundant and accurate high-level semantic information in images.

### 4.4. Visualization Analysis

To show the ability to localize visual concepts, we extracted and visualized the feature maps from the last convolutional layer of the MIL header module as the heat maps. As shown in Figure 6, the proposed RUDet can correctly locate the visual concepts corresponding to both nominal words and non-nominal words. With the help of the effective attributes detected by our method, the generated caption sentences are reasonable and close to the ground truth captions.

## 5. Conclusions

In this paper, we presented our approach of Refined Universal Detection (RUDet), which can detect effective attributes from images, especially for non-nominal words and similar words. In the proposed method, a Refinement (RF) module is designed to learn refined attributes for non-nominal words based on the knowledge of the initial attributes from the base model and image visual features. Besides, a Word Tree (WT) module is constructed, which ensures similar, reasonable, and more accurate probabilities for synonymous nominal words in same images. Furthermore, a Feature Enhancement (FE) module is employed to extract multi-scale visual features; thus, it helps to generate more accurate image attributes. Experiments indicated that our method can boost the performance of attribute detection significantly and outperform other existing methods. In addition, the attributes detected by our method help to improve the performance of state-of-the-art image captioning methods.

Technically, our method has the ability to detect attributes of various of concepts and can be applied in many other domains, such as visual question answering, video captioning, three-dimensional visual understanding, and so on. In the future, we will continue to improve the capability of our method and apply this method on other domains.

## Figures and Tables

**Table 1:** The comparison among the different versions of the proposed RUDet. In each column, the highest values are marked by **underline**.

					Word Count/Average Precision (AP)				
	Backbone	FE module	WT module	RF module	NN	VB	JJ	Others	All
	Word Count				616	176	119	89	1000
a)	ResNet50				41.08	22.06	26.69	21.47	34.28
b)	ResNet50	✓			42.30	22.94	27.72	22.24	35.37
c)	ResNet50		✓		42.81	23.10	27.67	22.02	35.69
d)	ResNet50			✓	42.35	22.66	27.43	21.92	35.29
e)	ResNet50	✓	✓	✓	44.13	24.03	28.63	22.89	36.85
a)	ResNet101				42.09	23.02	27.61	21.89	35.21
b)	ResNet101	✓			43.28	24.16	28.68	22.61	36.34
c)	ResNet101		✓		43.79	24.06	28.64	22.38	36.61
d)	ResNet101			✓	42.96	24.01	28.44	22.55	36.08
e)	ResNet101	✓	✓	✓	45.95	25.65	30.32	23.55	38.53
a)	ResNet152				42.31	22.90	27.24	21.82	35.28
b)	ResNet152	✓			43.46	23.91	28.28	22.52	36.35
c)	ResNet152		✓		43.99	23.82	28.08	22.29	36.62
d)	ResNet152			✓	43.21	24.06	27.89	22.54	36.17
e)	ResNet152	✓	✓	✓	**46.80**	**26.26**	**30.97**	**23.85**	**39.26**

**Table 2:** The comparison of the proposed RUDet with other methods. For the proposed RUDet, “ResNet152” means using Residual Network with 152 layers (ResNet152) as backbone, and “+FE+WT+RF” means using the FE module, the WT module, and the RF module. In each column, the highest values are marked by **underline**.

		Word Count/Average Precision (AP)				
Method		NN	VB	JJ	Others	All
Word Count		616	176	119	89	1000
MIL (AlexNet) [3]		36.90	18.00	22.90	19.90	30.40
MIL (VGG) [3]		41.40	20.70	24.90	21.23	34.00
MAD [7]		37.72	17.26	22.16	9.43	30.07
MAD-retrained [7]		40.60	20.84	25.38	20.82	33.55
RUDet (ResNet50)	(ours)	41.08	22.06	26.69	21.47	34.28
RUDet (ResNet152+FE+WT+RF)	(ours)	**46.80**	**26.26**	**30.97**	**23.85**	**39.26**

**Table 3:** The effect on the Average Precisions (APs) of synonyms (“phone” and “cellphone”, “laptop” and “computer”) by the WT module. The *difference* is calculated by subtracting the APs of two synonymous words; the **lower**↓, the better. The *mean* is the mean value of the APs of two synonymous words; the **higher**↑, the better.

	Average Precision (AP)	
Word	Without WT module	With WT module
phone	69.95	71.14
cellphone	41.34	45.12
(*difference*)	28.61	**26.02**↓
(*mean*)	55.65	**58.13**↑
laptop	97.27	95.79
computer	85.81	87.86
(*difference*)	11.46	**7.93**↓
(*mean*)	91.54	**91.83**↑

**Table 4:** The comparison with other methods in caption generation. In the first column, “RUDet+” means using the attributes detected by the proposed RUDet. The symbol “–” means that the authors did not provide the values of the corresponding metrics. In each column, the highest values are marked by **underline**, and other values improved by our method are marked by dashed underline.

Methods		BLEU-1	BLEU-2	BLEU-3	BLEU-4	Meteor	ROUGE-L	CIDEr-D
LSTM-A [4]		73.4	56.7	43.0	32.6	25.4	54.0	100.2
Att-R+LSTM [11]		74.0	56.0	42.0	31.0	26.0	–	94.0
MAD+SAP [7]		–	–	–	38.6	28.7	58.5	128.8
SCST [14]		–	–	–	34.2	26.7	55.7	114
Up-Down [15]		79.8	–	–	36.3	27.7	56.9	120.1
SGAE [28]		**81.0**	–	–	39.0	28.4	58.9	129.1
RUDet+LSTM-A	(ours)	**75.9**	**59.8**	**46.1**	**35.4**	**27.1**	**56.1**	**110.4**
RUDet+Att-R+LSTM	(ours)	**75.8**	**59.8**	**46.2**	**35.6**	**27.1**	**56.0**	**110.2**
RUDet+MAD+SAP	(ours)	**79.5**	**64.3**	**50.8**	**40.2**	**29.5**	**59.3**	**129.6**

## Data Availability

No new data were created or analyzed in this study. Data sharing is not applicable to this article.

## Abbreviations

The following abbreviations are used in this manuscript:

RUDet	Refined Universal Detection
FE	Feature Enhancement (module)
WT	Word Tree (module)
RF	Refinement (module)
MS COCO	Microsoft Common Objects in Context (dataset)
CNN	Convolutional Neural Network
MIL	Multiple Instance Learning
CAM	Class Activation Mapping
AP	Average Precision
POS	Part of Speech
FC	Fully Connected
FPN	Feature Pyramid Network
SPP	Spatial Pyramid Pooling
NN	nouns
VB	verbs
JJ	adjectives
